# Compounds Derived from Tryptophan Metabolism in *Torulaspora delbrueckii* CBS1146^T^ and *Zygosaccharomyces bailii* ATCC36947^T^

**DOI:** 10.3390/ijms26094301

**Published:** 2025-05-01

**Authors:** Alessandra Di Canito, Michele Dei Cas, Sara Vitalini, Marcello Iriti, Rita Paroni, Ileana Vigentini, Roberto Foschino

**Affiliations:** 1 One Health Unit, Department of Biomedical, Surgical and Dental Sciences, Università degli Studi di Milano, 20122 Milan, Italy; alessandra.dicanito@unimi.it (A.D.C.); sara.vitalini@unimi.it (S.V.); marcello.iriti@unimi.it (M.I.); roberto.foschino@unimi.it (R.F.); 2 Department of Health Sciences, Università degli Studi di Milano, 20142 Milan, Italy; michele.deicas@unimi.it (M.D.C.); rita.paroni@unimi.it (R.P.)

**Keywords:** kynurenic acid, kynurenine, melatonin, tryptophan, tryptophan-ethylester isomers, *Torulaspora delbrueckii*, *Zygosaccharomyces bailii*

## Abstract

Yeast metabolism significantly contributes to functional beverage production by generating bioactive compounds such as tryptophan derivatives (dTRPs). While *Saccharomyces cerevisiae* is traditionally used, non-Saccharomyces yeasts like *Torulaspora delbrueckii* and *Zygosaccharomyces bailii* are gaining interest for their ability to enhance aroma profiles and influence metabolite synthesis. This study evaluated the dTRP production of *T. delbrueckii* CBS1146^T^ and *Z. bailii* ATCC36947^T^ in synthetic medium and Cabernet Sauvignon must supplemented with 100 mg/L tryptophan. LC-MS/MS analysis revealed strain-dependent differences in metabolite profiles, with a predominance of kynurenine pathway compounds and the first identification of two tryptophan-ethylester (TEE) isomers. *T. delbrueckii* exhibited significant TEE production, correlating with the consumption of dTRPs; conversely, *Z. bailii* synthesized diverse metabolites, including 5OH-tryptophan and kynurenic acid. Notably, melatonin was not detected. The fermentation matrix strongly influenced dTRP biosynthesis, with must conditions enhancing TEE and kynurenic acid accumulation. These findings highlight the role of growth medium composition in modulating yeast metabolism and support the potential of non-*Saccharomyces* yeasts for functional beverage development.

## 1. Introduction

The requests of consumers and the availability on market of functional foods and beverages are significantly rising at this time, due to the increased attention of people for healthy eating and drinking [1,2]. Functional foods have been defined by the European legislation [3] as foods that positively impact body functions beyond basic nutrition, improving health or reducing disease risk. In particular, fermented materials have attracted considerable attention due to their beneficial effects on human health [4,5,6]. Indeed, their potential can be linked to the metabolism of microorganisms that, when proliferating in different matrices, also lead to the synthesis of neuroactive compounds [7,8,9]. Growing interest has been given to vegetable fermented beverages as a source of bioactive molecules (i.e., vitamins, essential fatty acids, amino acid derivatives), that together with polyphenols, flavonoids, and carotenoids can support the immune system, helping in weight management, improving gut and cardiovascular health or acting as an adjuvant to counteract the aging processes [10,11].

In many cases fermentative processes are carried out by yeasts able to use several different raw materials to produce alcoholic beverages (wine, beer, cider and fruit wines), fermented cereal-based doughs and dairy products [12,13]. These biological transformations are traditionally conducted by *S. cerevisiae* strains; however, manufacturing companies recently decided to select non-*Saccharomyces* yeasts including *Torulaspora delbrueckii* and *Zygosaccharomyces bailii* [14,15]. Actually, non-*Saccharomyces* yeasts can play a significant role in winemaking in terms of aroma profile through the production of volatile compounds such as short-chain fatty acids, higher alcohols and relevant esters, as well as the generation of bioactive molecules [16,17]. Among published papers, in Fracassetti et al. (2019) [18], a *T. delbrueckii* strain was used to obtain an innovative kiwi-based wine like that obtained by Li et al., 2022 [19] with a *Z. bailii* strain. Moreover, *T. delbrueckii* species is widely studied for its contribution to the wine flavor [20], while *Z. bailii* has been found to be a dominant species in the spontaneous Maotai liquor fermentation [21]). Furthermore, in 2017, Freire and collaborators described the potentialities of a *T. delbrueckii* strain to produce a non-dairy fermented beverage increasing its antioxidant properties [22]. The great potential of yeast metabolites in functional beverages production is confirmed by the presence of biochemical pathways for the synthesis of useful molecules deriving from tryptophan (TRP) such as melatonin (MEL) and serotonin (5 OH TRY) [23,24].

TRP is a non-polar, aromatic amino acid containing an indole ring and after its absorption can be converted into bioactive compounds, each able to influence metabolic pathways and physiological responses [23]. Two pathways are involved in TRP transformation; the first leads to the breakdown of the indole ring forming kynurenine (KYN) and its derivatives, the second one preserves the indole ring producing chemical messengers of the indolamine family, among which is melatonin (MEL) [25]. It was demonstrated that some fermented foods contain KYN, kynurenic acid (KYNA) and MEL [26,27]. The KYN pathway has been characterized in starter yeast species such as *S. cerevisiae* and *Saccharomyces uvarum*, used in manufacturing bakery products and beer [28,29] and it has been suggested that this pathway may be essential for the detoxification of TRP excess by forming the less toxic kynurenic acid (KYNA) [30]. Noteworthy, the human KYN pathway is involved in neurodegenerative and autoimmune disorders (Alzheimer’s disease, Huntington’s disease, amyotrophic lateral sclerosis), but also in carcinogenesis and psychiatric syndromes [31,32,33]. In particular, KYNA is considered cytoprotective, in addition to its antioxidant and neuroprotective properties, while other metabolites are considered with cytotoxic/pro-epilepsy effect, like 3OH kynurenine (3 OH KYN), 3OH anthranilic acid (3OH AA), quinolinic and nicotinic acid [23]. Finally, Widner and collaborators in 2002 [34] found that a higher ratio of KYN/TRP revealed to be a consistent marker for the Parkinson’s disease.

The less common route of TRP transformation in yeasts is the pathway leading to MEL synthesis; different reactions can be involved: (i) acetylation of 5OH TRY to N-acetyl serotonin (NAC 5OH TRY); (ii) 5OH TRY conversion to MEL through 5-methoxytryptamine (5OME TRY) [35,36]. Nevertheless, the first step of the pathway is the decarboxylation of TRP to tryptamine (TRY) [37], suggesting the existence of diverse MEL biosynthesis pathways in *S. cerevisiae*. In fact, only one gene has been identified in *S. cerevisiae* to be involved, the *PAA1* gene, which codes for a polyamine acetyltransferase [38]. However, MEL does not exist only in the form of N-acetyl-methoxindolamine; indeed, structural isomers have been discovered, and considering the position of the two side chains connected to the indole ring, 42 combinations have been calculated [39]. Surprisingly, these isomers can have diverse biological functions, such as antioxidant and cytoprotective activity depending on the position of the two side chains in the indole ring [40,41,42]. Tryptophan derivatives (dTRPs) are used in a wide range of industries, including the chemical, food, polymer and pharmaceutical sectors. Moreover, dTRPs play a key role in treating disease and improving quality of life. Their versatility and wide range of uses make them valuable components in various industrial processes and therapeutic interventions [43]. Despite the growing interest in non-*Saccharomyces* yeasts for food and beverage production, no studies have been carried out to evaluate their ability to produce bioactive compounds. The aim of this work was to investigate the synthesis of dTRPs by *T. delbrueckii* and *Z. bailii* strains under laboratory conditions to explore their potential application in fermented functional foods.

## 2. Results and Discussion

*T. delbrueckii* CBS1146^T^ and *Z. bailii* ATCC36947^T^ strains were chosen for the experiments of growth and production of dTRPs. These strains had been previously investigated for the production of melatonin isomers and tryptophan ethyl ester (TEE) in different musts [44]. Particularly, the KYN pathway was monitored throughout the detection of KYN, 3OH KYN, KYNA, AA and 3OH AA. On the other hand, the content of 5OH TRP, serotonin (5OH TRY), NAC 5OH TRY, MEL and its potential isomers and TEE was quantified to follow the MEL biosynthesis route. The trials have been performed setting up a liquid cultural medium containing 100 mg/L TRP as precursor and in the same conditions described in Dei Cas et al. (2021) [23], since no abiotic degradation of TRP was observed.

Similarly, the strains were subjected to fermentation tests in real grape must to evaluate their behavior in a widespread material for the production of beverages.

The release of dTRPs by cells is crucial for metabolic regulation, intercellular communication and detoxification. In yeasts, specific transporters or vesicular mechanisms export dTRPs, including KYN, a key intermediate in TRP degradation. KYN regulates metabolism by modulating TRP degradation flux in response to environmental changes or metabolic demands. It also acts as a signaling molecule, influencing intercellular communication, symbiosis or competition. Additionally, KYN export facilitates detoxification by removing excess TRP or toxic metabolites, promoting survival in diverse ecological niches and microbial ecosystems [45]. On the other hand, excessive indole may also inhibit the activity of tryptophanase TnaA and the transport process of tryptophan [43].

Moreover, the importance of TRP and dTRPs in dietary to maintain health state and promote human well-being is widely recognized.

### 2.1. Release of dTRPs by T. delbrueckii CBS1146^T^

**Figure 1:**
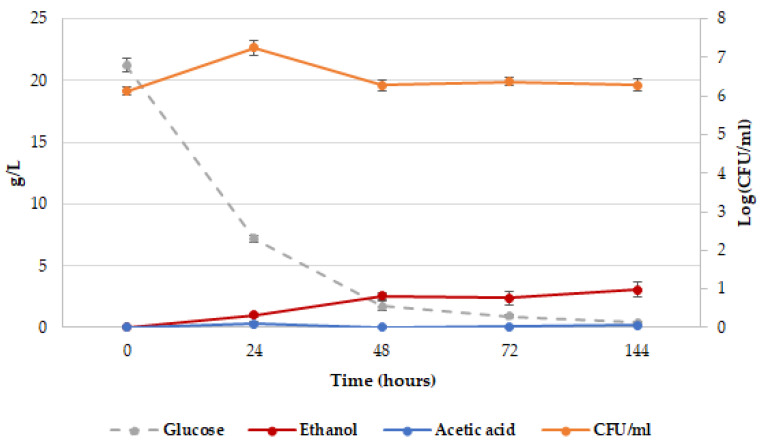
*T. delbrueckii* CBS1146^T^ kinetics in YNBT100 medium at 25 °C. Plate counts (Log CFU/mL), orange line; glucose concentration, gray line; ethanol concentration, red line; acetic acid concentration, blue line. The reported data are the average of three replicates, and the bars indicate the standard deviations.

The plate counts of *T. delbrueckii* CBS1146^T^ in YNBT100 medium, the related consumption of glucose and production of acetic acid and ethanol during the fermentation are shown in Figure 1. In the first 24 h of incubation, the yeast cells reached the highest biomass level (1.8 × 10^7^ CFU/mL), consuming the most part of the glucose; a decrease in the cell population was detected after 48 h (1.9 × 10^6^ CFU/mL), and it was followed by a plateau level up to the end of the fermentation. Concerning the acetic acid, the highest production was observed at 48 h (0.14 ± 0.01 g/L), and it remained almost stable till the end of the process; on the contrary, the ethanol level continued to increase up to the last time of sampling (3.06 ± 0.01 g/L).

**Figure 2:**
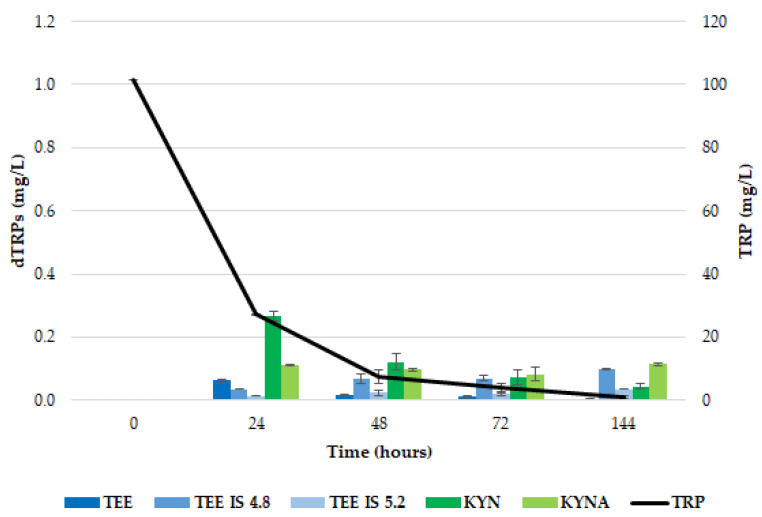
Production of dTRPs in *T. delbrueckii* CBS1146^T^ in YNBT100 medium at 25 °C. The black line is referred to the TRP consumption, while the histograms are referred to each identified compound (TEE and its isomers in blue; KYN and KYNA in green). The reported data are the average values of three replicates, and the bars indicate the standard deviations.

In Figure 2, the production of the dTRPs by *T. delbrueckii* CBS1146^T^ in comparison with the TRP consumption is described. Most of the available TRP was used by the cells in the first 48 h of proliferation (about 93%), and it was exhausted during the fermentation.

**Figure 3:**
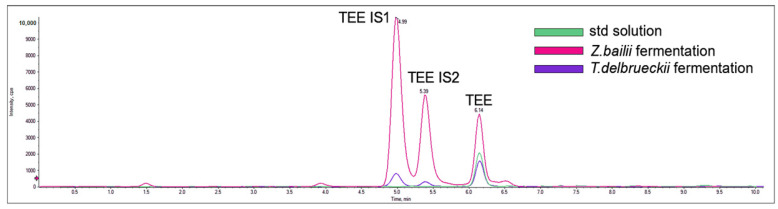
Melatonin isomers production by *T. delbrueckii* CBS1146^T^ and *Z. bailii* ATCC36947^T^. Chromatograms derive from an overlay of a standard pure solution containing all the indoles analyzed at a concentration of 2 ng/mL (green trace) and samples of *T. delbrueckii* CBS1146^T^ (purple trace) and *Z. bailii* ATCC36947^T^ (turquoise trace) after fermentation of 24h. TEE (rt 6.11, *m*/*z* 233 > 216) and its isomers, namely TEE IS1 (rt 4.99, *m*/*z* 233 > 216) and TEE IS2 (rt 5.39, *m*/*z* 233 > 216), were shown.

Considering the MEL biosynthesis route, unlike what was described in Dei Cas et al. (2021) [23] for *S. cerevisiae* EC1118, neither the searched metabolites (5OH TRP, 5OH TRY and NAC 5OH TRY) nor MEL were detected in the supernatant, probably due to a rapid conversion of MEL in the other indoles [36], as observed by Muniz-Calvo and co-authors (2019) [37] who verified MEL production in *S. cerevisiae* only after the supplementation of 5OH TRP and not that of TRP. The kinetics of TEE production showed an accumulation peak at 24 h, corresponding to the highest extracellular release (0.063 ± 0.003 mg/L), followed by a rapid decrease in its amount (Figure 2). Two TEE isomers were detected, showing 4.99 min (TEE IS1) and 5.39 min (TEE IS2) retention times; for both, the highest accumulation was observed at the end of the fermentation, corresponding to 0.099 ± 0.002 mg/L and 0.036 ± 0.002 mg/L, respectively (Figure 3). Since these isomers, which have the same mass spectrometric behavior as TEE (*m*/*z* 233 > 216) but different retention times on chromatography (Rt 4.99 and 5.39 min instead of Rt 6.11 min), only occur in the sample with yeast growth, it can be concluded that they were produced by using tryptophan. Gardana et al. (2014) [46] first discovered the presence of a tryptophan-ethylester (TEE) in wine, having the same molecular weight of MEL. Subsequently, Vigentini and collaborators (2015) [44] confirmed this observation in fermentation trials driven by different *S. cerevisiae* and non-*Saccharomyces* yeast strains. It has been speculated that TEE could have an advantage on the precursor (probably TRP) because of its more lipophilic structure, so it can easily cross cell membranes and be metabolized to MEL in enteroendocrine cells of the gastrointestinal mucosa. Indeed, studies related to the Hartnup disease in children revealed that TEE administration modified TRP deficiency state, and in vitro experiments demonstrated that ester was hydrolysed by the intestinal mucosa, liver and kidney to provide TRP [47].

As concerns the KYN pathway, the accumulation trend of both KYN and KYNA was comparable with that observed for TEE, starting in the first 24 h of growth reaching the concentrations of 0.267 ± 0.014 and 0.110 ± 0.0017 mg/L, respectively. Then, KYN started to be metabolized, reducing its content to around 83.5% after 144 h. On the contrary, KYNA concentration remained stable till the end of the experiment (0.114 ± 0.003 mg/L). Unlike what occurs in *S. cerevisiae* EC1118, where KYNA shows a positive accumulation trend along the fermentation [23], in *T. delbrueckii*, an accumulation plateau for KYNA was observed, suggesting that KYN conversion could lead to other intermediates. Another possibility could be the inhibition of kynurenine aminotransferase, the enzyme leading to KYNA from KYN. In any case, metabolites linked to the KYN pathway (3OH KYN, 3OH AA and AA) failed to be detected, although molecules other than those we searched for may have increased, such as picolinic acid and xanthurenic acid [48].

Consequently, these results suggested, as described by O’Connor-Cox et al. in 1989 [46], that a great part of TRP was probably metabolized as a nitrogen source for the cell growth and biomass production, being essential for protein synthesis.

In order to deduce a possible linear relationship between the levels of the five dTRPs detected, the Pearson correlation coefficient (r) was calculated (Table 1). The results of the analysis showed, as expected, a significant negative correlation between TRP and its derivatives (except for KYN). In particular, TEE_IS1, TEE_IS2 and KYNA exhibited high negative coefficients with TRP (−0.9007, −0.8600 and −0.8943, respectively), indicating that an increase in their concentration is accompanied by a decrease in the amino acid content from which they derive. TEE showed a positive correlation with KYN (0.9747), suggesting a tendency for these compounds to change in the same direction, probably due to their consumption to produce TEE isomers and KYNA, respectively. Indeed, both the TEE isomers revealed a positive correlation with KYNA, as well as each other, that could reveal coordinated changes under physiological states. We do not know the chemical structures of TEE isomers, but different physicochemical properties and functional role in yeast cells might explain the higher levels of TEE_IS1 with respect to TEE_IS2.

### 2.2. Release of dTRPs by Z. bailii ATCC36947^T^

**Figure 4:**
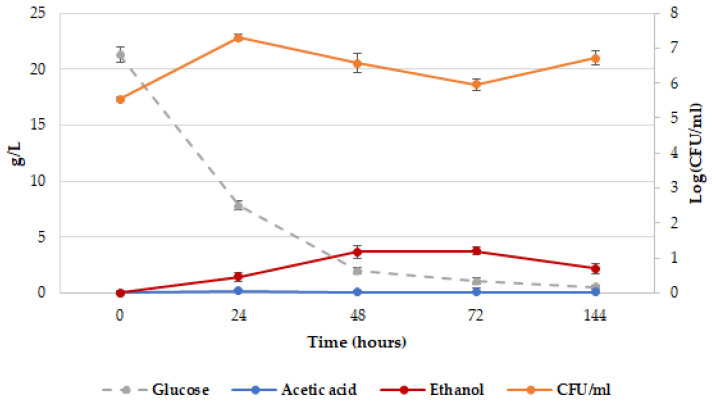
*Z. bailii* ATCC36947^T^ kinetics in YNBT100 medium at 25 °C. Plate counts (Log CFU/mL), orange line; glucose concentration, gray line; ethanol concentration, red line; acetic acid concentration, blue line. The reported data are the average of three replicates, and the bars indicate the standard deviations.

Figure 4 shows the plate counts of *Z. bailii* ATCC36947^T^ in YNBT100 with the relevant consumption of glucose and production of acetic acid and ethanol.

Up to 24 h of incubation, the yeast strain grew, reaching the highest biomass level (2.0 × 10^7^ CFU/mL) and consuming most of the glucose. After 72 h, the viable cell concentration decreased to 8.9 × 10^5^ CFU/mL, followed by an increase at the end of the experiment (5.2 × 10^6^ CFU/mL at 144 h).

The acetic acid content remained low and stable during the fermentation process, while the level of ethanol increased, attaining the highest value after 72 h (3.76 ± 0.01 g/L), and then it slightly decreased till the end of the fermentation. This finding can be explained by the fact that it was consumed for a partial regrowth of the strain’s cells.

**Figure 5:**
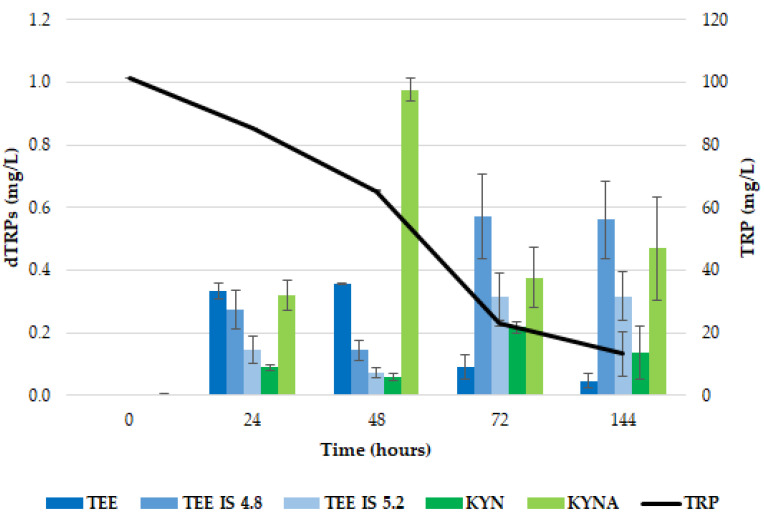
Production of dTRPs in *Z. bailii* ATCC36947^T^ in YNBT100 medium at 25 °C. The black line refers to the TRP consumption, while the histograms refer to each identified compound (TEE and its isomers in blue; KYN and KYNA in green). The reported data are average values of three replicates, and the bars indicate the standard deviations.

In Figure 5. the dTRPs synthesis by *Z. bailii* ATCC36947^T^ linked to the TRP consumption is shown. Yeast cells consumed about 77.4% of the available TRP within 72 h of proliferation.

Considering the MEL biosynthesis pathway, in *Z. bailii* ATCC36947^T^, the metabolites 5OH TRP, TRY, 5OH TRY and NAC 5OH TRY and MEL ISO4.8 were detected in traces in the supernatants (Table 2). Particularly, MEL was not detected, confirming the results obtained by Dei Cas et al., 2021 [23], for *S. cerevisiae* EC1118. At the same way, we observed a similar accumulation trend of 5OH TRY and TEE up to 48 h, when the highest concentration was detected (0.355 ± 0.002 mg/L for TEE); later, a rapid decrease in the molecule amount was found, and the two TEE isomers, already noticed in *T. delbrueckii* fermentations, were observed (Figure 5). Their highest build-up was detected after 72 h, corresponding to 0.572 ± 0.134 mg/L and 0.314 ± 0.076 mg/L, for TEE IS1 and TEE IS2, respectively. These concentrations remained stable until the end of the fermentation (144 h).

Concerning the KYN route, the accumulation of KYN and KYNA followed a different trend. The highest level of KYN was detected at 72 h (0.265 ± 0.020 mg/L), while for KYNA it was recorded at 48 h (0.975 ± 0.038 mg/L), similarly to what was observed for TEE. Then, KYN content was reduced of 38.5% at the end of the fermentation, while KYNA concentration decreased to 56.7% immediately after the maximum accumulation, revealing a different behavior from *S. cerevisiae* EC1118 [23]. It could be probably degraded to synthetize other metabolites. Indeed, other molecules linked to the KYN pathway (3OH KYN, 3OH AA and AA) were detected, even if just in traces that were absent in *T. delbrueckii* CBS1146^T^ (Table 2).

As already mentioned, these results confirmed the hypothesis of O’Connor-Cox et al. in 1989 [49].

The Pearson correlation coefficient (r) was calculated also for the dTRPs produced by *Z. bailii* ATCC36947^T^. As expected, the results revealed a significant negative correlation for TRP with the TEE_IS1 and TEE_IS2 isomers, KYN and MEL IS0 4.8 (−0.7434, −0.7222, −0.7184 and −0.7436, respectively) (Table 3), indicating a possible relationship within the metabolic pathway. On the contrary, several metabolites displayed strong positive correlations with each other. For example, TEE with AA (0.7152) and TRY (0.6963), and TEE isomers with KYN, 5OH-TRP and MEL ISO 4.8, suggesting a tendency for these compounds to change together. Concerning the metabolites of the kynurenine pathway, KYN was positively correlated with 5OH-TRP and MEL ISO 4.8, while KYNA and AA showed a positive association with all the metabolites of the route, except for MEL ISO 4.8. Finally, positive correlations were observed for 3OH-AA vs. NAC- 5OH TRY, 5OH-TRY vs. TRY and NAC-5OH TRY.

The correlation patterns observed in Table 3 reveal important insights into the metabolic dynamics of *Z. bailii* ATCC36947^T^. The negative correlations between TRP and downstream metabolites like TEE isomers, KYN and MEL ISO4.8 indicate active consumption of the precursor amino acid and its conversion into these derivatives during fermentation. Notably, the strong positive correlations among certain metabolites highlight key metabolic relationships: the association between TEE and both AA and TRY suggests parallel processing of TRP through esterification and decarboxylation pathways. Meanwhile, the tight linkage between KYN and both 5OH-TRP and MEL ISO4.8 points to coordinated regulation between the KYN and serotonin/MEL biosynthetic routes, likely mediated by shared enzymatic machinery. The interconnectedness of the KYN pathway is further evidenced by the positive associations of KYNA and AA with most pathway intermediates. These correlation patterns reflect *Z. bailii*’s distinct metabolic architecture, which favors TRP flux through the KYN pathway over MEL synthesis, consistent with previous observations in non-*Saccharomyces* yeasts [23]. The robust correlation between the two TEE isomers additionally suggests they may be co-produced through similar enzymatic mechanisms, though their specific biochemical origins warrant further investigation.

### 2.3. Different Behavior of the Analyzed Yeasts

**Figure 6:**
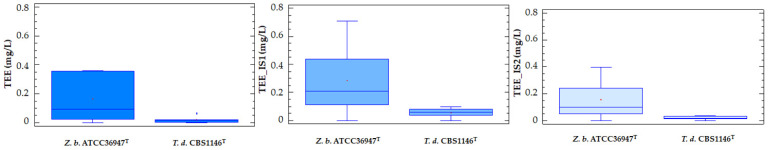
Box plots of the data concerning the production of TEE isomers by the two strains.

Concentration values of each detected compounds were subjected to one-way ANOVA analysis to investigate the potential single effects of strain. Concerning the TEE and its isomers, the accumulation of these metabolites was significant different (*p* < 0.01) between them and, specifically, *Z. bailii* ATCC36947^T^ proved to be the highest producer in terms of yield (mg/L) (Figure 6). Averages of TEE were 0.0191 mg/L and 0.1655 mg/L (*p* = 0.0012) for *T. delbrueckii* CBS1146^T^ and *Z. bailii* ATCC36947^T^, respectively. Moreover, the isomers TEE_IS1 and TEE_IS2 mean levels were of 0.0545 mg/L and 0.0189 mg/L for *T. delbrueckii* CBS1146^T^ while reaching 0.2857 mg/L and 0.1547 mg/L for *Z. bailii* ATCC36947^T^.

**Figure 7:**
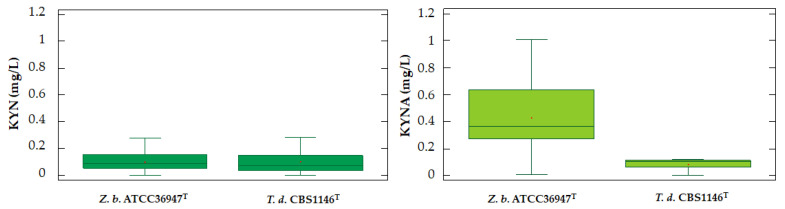
Box plots of the data concerning the production of KYN and KYNA compounds by the two strains.

Also, the trends of KYN and KYNA productions were different, confirming what observed by Yilmaz and collaborators in 2019 [50]. These authors suggested that the intermediate productions were linked to the strains and the growth conditions, assessing them in *S. pastorianus* and *S. cerevisiae*. Indeed, at the moment in which *T. delbrueckii* CBS1146^T^ started to produce the metabolites, the amount of the precursors decreased till the end of the fermentation process; on the contrary, this situation was not observed in *Z. bailii* ATCC36947^T^ which started to synthetize over KYN after 72 h of fermentation. The ANOVA results revealed significant differences (*p* < 0.01) for KYNA synthesis confirming *Z. bailii* ATCC36947^T^ as the highest producer in terms of yield (mg/L) for this compound. Particularly, the average amounts were 0.0806 mg/L for *T. delbrueckii* CBS1146^T^ and 0.4289 mg/L for *Z. bailii* ATCC36947^T^. On the other hand, the quantities of KYN were not statistically different (*p* = 0.9859) with mean values of 0.1009 mg/L and 0.1003 mg/L for *T. delbrueckii* CBS1146^T^ and *Z. bailii* ATCC36947^T^, respectively (Figure 7). The lack of significant differences in KYN levels may be attributed to its role as a central intermediate in the pathway, which is tightly regulated in yeast metabolism. Both strains likely convert TRP to KYN efficiently, but downstream flux diverges based on species-specific enzymatic activities (e.g., kynurenine aminotransferase in *Z. bailii* favoring kynurenic acid (KYNA) production). This consistency in KYN accumulation, despite differing metabolic outputs, underscores its pivotal position in TRP catabolism.

### 2.4. Accumulation of dTRPs in Grape Must

Growth tests were conducted with both yeast strains in Cabernet Sauvignon must, supplemented with 100 mg/L of TRP, to examine its fate in derivatives under similar oenological conditions. The initial TRP concentration in the must was 2.7 mg/L. Fermentations were carried out at 25 °C under static conditions and monitored daily by weight loss measurements due to carbon dioxide evolution. Table 4 presents yeast counts, glucose, fructose, and tryptophan consumption, along with ethanol, acetic acid, and dTRPs production from the beginning to the end of incubation. Both strains exhibited significant growth, increasing cell concentration by more than one order of magnitude, and they consumed the sugars present without fully depleting them. Due to their differing alcohol tolerances, fermentation slowed to 11 days at approximately 10% ethanol (*v*/*v*) for *T. delbrueckii* and 8.5% (*v*/*v*) for *Z. bailii*. Both yeasts produced small quantities of acetic acid. *T. delbrueckii* utilized 63% of the available TRP, while *Z. bailii* consumed it entirely. TRP uptake in *Saccharomyces cerevisiae* is regulated by permeases such as Tat1p, Tat2p and Gap1p, which adjust amino acid transport based on extracellular availability and intracellular demand [51], while the secretion of dTRP remains less characterized but appears to occur via passive diffusion or specialized transport systems, including ATP-binding cassette (ABC) transporters and major facilitator superfamily (MFS) proteins [52]. These transporters play a crucial role in the excretion of secondary metabolites, including aromatic amino acid derivatives. The secretion of dTRPs may also have physiological relevance in microbial ecosystems, as these compounds can serve as signaling molecules or precursors for bioactive metabolites [53].

A key observation was the strain-dependent production and utilization of KYN. This metabolite is indeed a key intermediate in the TRP degradation pathway, contributing to NAD^+^ biosynthesis, which is essential for cellular metabolism [28]. However, under specific conditions, such as nutrient limitation or overflow metabolism, yeast cells may excrete KYN to regulate intracellular metabolite balance, prevent potential cytotoxic effects, or modulate interactions with other microorganisms in a competitive environment [54]. A distinct KYN metabolic flux was observed in the two strains: *T. delbrueckii* released it, while *Z. bailii* consumed it. Unexpectedly, KYNA concentration increased a hundredfold compared to synthetic medium tests, along with 3OH-AA and 3OH-KYN. The increased levels suggest highly active downstream metabolic pathways, likely influenced by ethanol accumulation [27]. Additionally, *Z. bailii* increased TRY concentration fivefold, while *T. delbrueckii* produced NAC-TRY. These results indicate yeast-specific TRP metabolism responses to nutrient availability and physiological stress [44].

Moreover, both strains synthesized TEE and its isomers at significantly higher levels than in YNBT100 medium. Melatonin was never detected.

The results support prior findings by Vigentini et al. [44], showing that must composition redirects TRP metabolic fluxes away from melatonin synthesis but generates substantial amounts of TEE and its isomers. Similarly, Yılmaz and Gökmen [55] identified kynurenic acid formation in white wine fermented with *T. delbrueckii, K. thermotolerans,* and *S. cerevisiae*, while no previous research has explored dTRP production by *Z. bailii*. They also confirmed that TEE and its isomers significantly increased during alcoholic fermentation.

These results confirm that ethanol and the complex composition of grape must have strong influences, likely triggering stress responses distinct from synthetic medium conditions. Grape must, due to its high nutrient content, enhances yeast proliferation and metabolism, increasing dTRP production compared to synthetic media.

The unique behaviors of *T. delbrueckii* and *Z. bailii* suggest potential applications in modulating TRP metabolism during fermentation, with implications for producing functional fermented beverages enriched in bioactive dTRPs.

**Figure 8:**
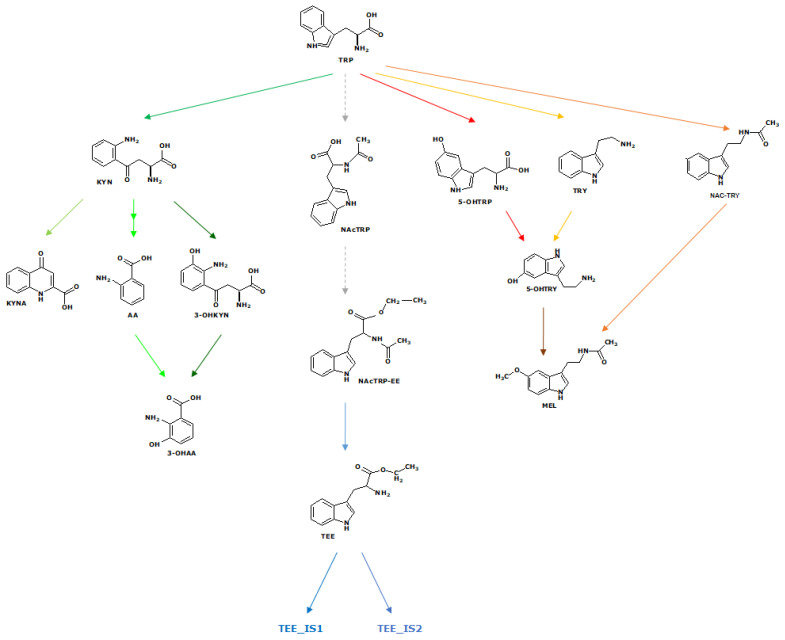
Biosynthetic pathways of the identified dTRPs. The colored arrows are associated with the detected metabolites.

In consideration of the results obtained in the must, it is feasible to delineate the plausible metabolic pathways for the two investigated strains. In Figure 8, the potential pathways of the identified metabolites are reported, highlighting the species on which they are identified.

## 3. Materials and Methods

### 3.1. Yeast Strain

The yeast strains used in this work were *T. delbrueckii* CBS1146^T^ (Westerdijk Institute, Utrecht, The Netherlands) and *Z. bailii* ATCC36947^T^ (ATCC; Manassas, VA, USA). The pure cultures are maintained in glycerinated stock at −80 °C composed by YPD medium [10 g/L yeast extract, 20 g/L peptone, 20 g/L glucose (*w*/*v*)] with 20% (*v*/*v*) glycerol.

### 3.2. Growth Conditions in YNB-Based Media

Both strains were thawed by the glycerol stock, inoculated in 5 mL of YPD medium, and incubated at 25 °C for 24 h to collect the cells in the exponential growth phase (about 2–4 OD_600nm_). 1% (*v*/*v*) of each pre-inoculum was inoculated in 5 mL of YNB-based medium without amino acids [20 g/L glucose; 6.7 g/L YNB without amino acids, Difco, Heidelberg, Germany; pH 5.4] (YNBT20) at 25 °C for 72 h. Then, the cells were centrifuged at 4000× *g* (Hettich Zentrifugen, Rotina 380r, Tuttlingen, Germany) for 15 min, washed with sterile distilled water (sdH_2_O) and further centrifuged under the above-mentioned conditions. Each cell pellet was resuspended in 5 mL of YNB medium without amino acids and glucose [6.7 g/L YNB without amino acids, pH 5.4] for 24 h to set up starvation conditions. After a centrifugation at 4000× *g* for 15 min, the cells were resuspended in 2 mL of sdH_2_O.

The same basal medium added by 100 mg/L TRP (YNBT100), used as a precursor, was utilized to monitor the production of TRP derivatives (dTRPs) by growth trials.

The growth tests were set up in sterile 6-well plates with 10 mL of YNBT100 medium at 25 °C, 120 rpm (Stuart SSL1, Chelmsford, Essex, UK), inoculating the strains at 0.1 OD_600nm_ per well of each pre-inoculum. The plates were sealed up with parafilm and covered with aluminum foil to avoid the oxidative photodegration of the dTRPs. The monitoring of the growth was performed by cell plate counting in WL nutrient agar medium (Scharlab, Sentmenat, Barcelona, Spain); both the growth and the dTRPs production were evaluated at 0, 24, 48, 72 and 144 h after inoculation. The analysis was carried out in triplicate on each biological replicate. The fermentations were monitored determining the concentrations of glucose, acetic acid and ethanol by enzymatic assays using R-Biopharm (Darmstadt, Germany) kits, according to the manufacturer’s instructions.

### 3.3. Growth Conditions in Grape Must

Yeast pre-cultures of the two strains were prepared in YNB medium (Difco™, ThermoFischer Scientific, Waltham, MA, USA) supplemented with 20 g/L glucose. Incubation took place at 25 °C and 120 rpm (SSL1 Orbital Shaker, Stuart Scientific, Staffordshire, UK). After 72 h, cells were harvested by centrifugation at 4000× *g* for 15 min (Hettich, ROTINA 380R, Tuttlingen, Germany), followed by two washes with sterile distilled water. The resulting cells were then used to inoculate at a final concentration of 1 × 10^6^ CFU/mL a Cabernet Sauvignon must. The Cabernet Sauvignon must was collected and stored at –20 °C. Prior to use in this study, it was thawed, supplemented with 70 mg/L of potassium metabisulfite, and maintained at 4 °C. Experiments were conducted in 100 mL flasks filled with 30 mL aliquots, sealed with Muller valves (containing 10% (*v*/*v*) HCl) to create oxygen-limited conditions, and maintained at 25 °C ± 2 °C without agitation into the dark, and covered with aluminum foil to avoid the oxidative photodegration of the dTRPs. The flasks were weighed every 24 h to assess CO_2_ loss. Fermentations were conducted in triplicate. Plate counts (CFU/mL) were performed at the time of inoculation (0 days) and at the end of fermentation; after appropriate dilution in Peptone Water (peptone, 10 g/L; sodium chloride, 5 g/L, pH 7.2) (Merck, Darmstadt, Germany), samples were plated on YPD medium supplemented with 18 g/L agar (Scharlau, Barcelona, Spain) and incubated at 25 °C for three days. Additionally, a non-inoculated control flask was included to monitor potential contamination. Since no signs of fermentation or microbial growth were detected in the control, the potential presence of viable microbial cells was considered negligible. Finally, supernatants were collected, stored at −20 °C in the absence of light to prevent metabolite degradation, and subjected to chemical analysis to monitor fermentation progress, including glucose, acetic acid, and ethanol concentrations, as well as the presence of dTRPs.

### 3.4. TRP Conversion Tests

In the case of growth in YNB broth added by 100 mg/L TRP, the determination of TRP and dTRPs content was performed at 0, 24, 48, 72 and 144 h after the inoculation. One ml was collected at each sampling time from three wells of the sealed plates, centrifuged at 10,000× *g* for 10 min and the supernatants filtered with 0.22 µm filters for the LC-MS/MS analysis (stored at −20 °C).

### 3.5. Chemical Analysis of TRP Derivatives

In order to identify the dTRPs pure chemical standards (analytical grade) of TRY, NAC TRY, 5OH TRY, 5OME TRY, NAC 5OH TRY, 5F TRY, 5OH TRP, TRP, TRP D5, TEE, AA, 3OH AA, KYNA, KYN, 3OH KYN and MEL were used (Sigma-Aldrich; St. Louis, MO, USA). Moreover, the standard of the isotopic isomer of the MEL (MEL OCD3) was synthesized by Prof. Andrea Penoni (Department of Science and High Technology, University of Insubria, Varese, Italy).

To purify the dTRPs, methanol and formic acid (FA) were acquired from Sigma-Aldrich (Milan, Italy), while all the aqueous solutions were arranged using Milli-Q purified water (Millipore, Milan, Italy).

#### 3.5.1. Purification of Supernatants Deriving from Yeast Fermentation

In total, 10 µL of the collected supernatants deriving from fermentation was added to 50 µL of a mixture of internal standards (MIX IS) [20 ng of TRP D5, 1 ng of 5F TRY and 1.1 ng of MEL OCD3] and 100 µL of precipitating solution (methanol + 0.5% FA). Then, the mixtures were stirred by vortexing and centrifuged for 5 min at 12,000 rpm and finally 100 µL was dried under nitrogen. Lastly, the sample was resuspended in a solution of 50 µL of dH_2_O + 0.5% FA, and 10 µL was injected into LC-MS/MS. Sensitive solid-phase extraction for MEL was also implemented to investigate its presence in concentration less than 2 ng/mL. A total of 1 mL of the collected supernatants derived from fermentation was added to 50 µL of a mixture of internal standards (MIX IS) [20 ng of TRP D5, 1 ng of 5F TRY and 1.1 ng of MEL OCD3] and introduced in a SPE cartridge (STRATA X, 10 mg/1 mL, Phenomenex, Torrance, CA, USA) previously conditioned with water (1 mL) and methanol (1 mL). After being washed with a mixture of water/methanol (95:5 *v*/*v*, 1 mL) SPE was eluted with pure methanol (1 mL). The extracts were dried and resuspended in 50 µL of dH_2_O + 0.5% FA, and 10 µL was injected into LC-MS/MS.

#### 3.5.2. LC-MS/MS Analysis for the Detection of dTRPs

The analytical system consisted of an HPLC (Dionex 3000 UltiMate instrument; Thermo Fisher Scientific, USA) coupled to a tandem mass spectrometer (AB Sciex 3200 QTRAP; AB Sciex, Milano, Italy) equipped with an electrospray ionization TurboIonSpray source. The separation of the indolic compounds, including dTRPs, was achieved using a reversed-phase Zorbax Eclipse XDB-C18 analytical column (4.5 × 50 mm, 1.8 µm. A linear gradient was established by mixing eluent A (water + 0.1% formic acid) and eluent B (methanol + 0.1% formic acid), with the following elution profile: 0–1 min (20% B), 1–5 min (20–60% B), 5–7 min (60% B), 7.0–7.2 min (60–95% B), 7.2–8.2 min (95% B), 8.2–8.5 min (95–5% B), 10 min (5% B). The flow rate was 0.4 mL/min, and the column temperature was maintained at 40 °C.

The tandem mass spectrometer operated in positive electrospray ionization mode (ESI^+^), with an ion spray voltage of 5.5 kV and a source temperature of 500 °C. Nitrogen was used as the nebulizing gas (GS1: 55 psi), turbo spray gas (GS2: 60 psi) and curtain gas (30 psi), while the collision-activated dissociation (CAD) was set to a low level. Multiple reaction monitoring (MRM) mode was employed, with a dwell time of 60–70 ms for all transitions and analytes. Compound-dependent parameters were optimized via direct infusion, and nitrogen served as the collision gas for MS/MS experiments.

Quantitative analysis was performed by interpolating the peak area of each analyte with its corresponding internal standard (IS) and calibration curve. Additional analytical details, including the specific MRM transitions and calibration methodologies, can be found in Dei Cas et al. (2021) and other relevant references [23,50].

#### 3.5.3. Validation of the Analytical Method

The method performances [specificity, precision, accuracy, linearity, the limit of detection (LOD) and limit of quantification (LOQ)] were reported in full details in Dei Cas et al., 2021 [23]. The lowest concentration of analytes that produces a signal-to-noise ratio greater than 10 ranged from 2 to 50 ng/mL.

### 3.6. Statistical Analysis

The results were subjected to statistical analysis, and they are reported as mean ± standard deviation. MINITAB Statistical Software 19 (Pennsylvania State University, University Park, PA, USA) was used to perform the analysis of variance (One-way ANOVA) and significant differences (*p* < 0.05) were evaluated. Finally, mean separation was determined using the Fisher LSD Method and a confidence of 95%.

## 4. Conclusions

Fermentation is a cost-effective and traditional process that can improve both the nutritional and sensory properties of food. This study focused on the production of dTRPs by non-*Saccharomyces* yeasts, such as *T. delbrueckii* and *Z. bailii*, which show promise for use in functional beverages. Notably, fermentation resulted in a significant increase in kynurenic acid (KYNA), a neuroprotective compound with antioxidant and anticonvulsant properties. Additionally, the presence of TEE and its isomers was observed, which raises interesting questions regarding their absorption and neuroactive potential.

While the production of dTRPs by yeasts may involve higher downstream processing costs compared to chemical synthesis, it offers a confident natural method to enrich foods and beverages with bioactive compounds. The targeted production of specific dTRPs can be achieved through careful selection of precursors, inhibitors, strains and growth conditions, highlighting the biochemical flexibility of fermentation. Importantly, the study showed how medium composition significantly affects metabolic pathways, emphasizing the potential of yeast fermentation to increase dTRP concentrations and support the development of novel health-oriented beverages.

Future research should aim to optimize fermentation conditions to maximize both the yield and bioavailability of target compounds, while broadening the range of microbial strains and substrates explored. Integrating multi-omics approaches, such as metabolomics, transcriptomics, and proteomics, will be crucial to elucidate the biosynthetic pathways and regulatory networks operating within *Saccharomyces* and non-*Saccharomyces* consortia, and to enhance the production of bioactive molecules that contribute to the functionality of the final product, as already demonstrated for *Saccharomyces* strain consortia [56,57]. In parallel, efforts should focus on scaling up these bioprocesses for industrial applications, addressing challenges related to feasibility, regulatory compliance and their potential in the formulation of functional foods and nutraceuticals. Finally, given the complexity of microbial interactions in winemaking, it would also be of interest to investigate how dTRPs produced by non-*Saccharomyces* yeasts might influence the physiology of *S. cerevisiae* during fermentation, potentially enhancing stress tolerance or modulating other relevant traits.

## Figures and Tables

**Table 1:** Correlation coefficients found among the quantitative values of dTRPs in *T. delbrueckii* CBS1146^T^; significant values (*p* < 0.05) are highlighted in bold format.

	TRP	TEE	TEE_IS1	TEE_IS2	KYN	KYNA
TRP	1					
TEE	−0.1893	1				
TEE_IS1	**−0.9007**	−0.1562	1			
TEE_IS2	**−0.8600**	−0.1324	**0.9886**	1		
KYN	−0.3234	**0.9747**	−0.0279	−0.0133	1	
KYNA	**−0.8943**	0.4824	**0.7826**	**0.7939**	0.5888	1

**Table 2:** Production of dTRPs derivatives related to KYN (3OH KYN, 3OH AA and AA) and MEL (5OH TRP, TRY, 5OH TRY and NAC 5OH TRY, MEL ISO4.8) pathways. The reported data are average values of three replicates with the relevant standard deviations.

	dTRPs (mg/L)							
Time (h)	AA	3OH AA	3OH KYN	5OH TRP	TRY	5OH TRY	NAC-5OH TRY	MEL ISO 4.8
0	0.0 ± 0.0	0.0 ± 0.0	0.0 ± 0.0	0.0 ± 0.0	0.0 ± 0.0	0.0 ± 0.0	0.0 ± 0.0	0.0 ± 0.0
24	0.006 ± 0.002	0.002 ± 0.000	0.003 ± 0.002	0.006 ± 0.002	0.001 ± 0.001	0.010 ± 0.004	0.007 ± 0.000	0.274 ± 0.064
48	0.011 ± 0.002	0.011 ± 0.009	0.020 ± 0.002	0.003 ± 0.001	0.003 ± 0.001	0.015 ± 0.000	0.011 ± 0.002	0.144 ± 0.033
72	0.006 ± 0.001	0.002 ± 0.000	0.013 ± 0.002	0.007 ± 0.004	0.002 ± 0.001	0.004 ± 0.001	0.002 ± 0.000	0.572 ± 0.134
144	0.006 ± 0.003	0.001 ± 0.001	0.022 ± 0.013	0.007 ± 0.004	0.001 ± 0.000	0.012 ± 0.000	0.002 ± 0.001	0.439 ± 0.244

**Table 3:** Correlation coefficients found among the quantitative values of dTRPs in *Z. bailii* ATCC36947^T^; significant values (*p* < 0.05) are highlighted in bold format.

	TRP	TEE	TEE_IS1	TEE_IS2	KYN	KYNA	AA	3OH AA	3OH KYN	5OH TRP	TRY	5OH TRY	NAC-5OH TRY	MEL ISO 4.8
TRP	1													
TEE	0.2647	1												
TEE_IS1	**−0.7434**	−0.0869	1											
TEE_IS2	**−0.7222**	−0.1052	**0.9984**	1										
KYN	**−0.7184**	−0.0569	**0.9835**	**0.9792**	1									
KYNA	−0.2967	0.6576	0.1853	0.1700	0.2209	1								
AA	−0.2909	**0.7152**	0.2491	0.2288	0.2713	**0.9091**	1							
3OH AA	−0.0127	0.5219	−0.1305	−0.1482	−0.1237	0.6713	**0.7101**	1						
3OH KYNA	−0.6451	0.1443	0.5631	0.5646	0.5520	**0.7708**	**0.7058**	0.3937	1					
5OH TRP	−0.5570	0.1546	**0.8518**	**0.8461**	**0.8569**	0.2814	0.3449	−0.1445	0.5473	1				
TRY	−0.2192	0.6963	0.2101	0.1839	0.3107	**0.8391**	**0.7527**	0.4008	0.5246	0.4555	1			
5OH TRY	−0.2534	0.6965	0.0521	0.0265	0.0364	**0.8222**	**0.8298**	0.5636	0.5872	0.3225	**0.7200**	1		
NAC-5OH TRY	0.1981	**0.9422**	−0.0877	−0.1015	−0.0736	**0.7648**	**0.8183**	**0.7314**	0.3017	0.0425	0.6346	**0.7084**	1	
MEL ISO 4.8	**−0.7436**	−0.0868	1	**0.9984**	**0.9835**	0.1854	0.2493	−0.1301	0.5632	**0.8518**	0.2102	0.0522	−0.0876	1

**Table 4:** Mean values of plate counts, sugars, ethanol, acetic acid, TRP and dTRPs amounts during the fermentation test in Cabernet Sauvignon must at 25 °C for 12 days. The reported data are the average of three replicates with the relevant standard deviations.

	Cabernet Must	*T. delbrueckii* CBS1146^T^	*Z. bailii* ATCC36947^T^
Fermentation Time	Initial	Final	Final
Counts (log CFU/mL)	6.25 ± 0.2	7.9 ± 0.3	7.6 ± 0.4
Glucose (g/L)	106.12 ± 3.58	13.53 ± 1.35	54.99 ± 9.66
Fructose (g/L)	110.64 ± 9.19	26.30 ± 2.23	7.91 ± 0.68
Ethanol (ml/100 mL)	0.04 ± 0.01	10.00 ± 0.45	8.45 ± 1.67
Acetic acid (g/L)	0.56 ± 0.06	0.95 ± 0.25	0.61 ± 0.03
TRP (mg/L)	104.2 ± 16.7	38.7 ± 2.8	0.8 ± 0.6
5OH-TRP (mg/L)	18.9 ± 8.9	17.2 ± 9.0	9.5 ± 1.4
TEE (mg/L)	1.3 ± 0.3	30.5 ± 5.6	11.1 ± 0.8
TEE_IS1 (mg/L)	n.d.	24.0 ± 1.5	7.1 ± 0.9
TEE_IS2 (mg/L)	n.d.	9.7 ± 1.5	2.9 ± 0.5
KYN (mg/L)	63.0 ± 40.9	86.4 ± 0.6	6.5 ± 0.5
KYNA (mg/L)	61.2 ± 11.7	336.2 ± 11.0	299.2 ± 8.4
AA (mg/L)	7.5 ± 1.2	2.5 ± 1.1	3.3 ± 0.6
3OH AA (mg/L)	1.0 ± 0.6	26.8 ± 4.9	36.2 ± 1.5
3OH KYN (mg/L)	12.4 ± 1.3	28.3 ± 5.5	22.0 ± 2.9
TRY (mg/L)	5.1 ± 3.2	4.5 ± 2.7	24.6 ± 1.7
5OH TRY (mg/L)	14.1 ± 2.1	15.9 ± 4.0	7.0 ± 2.9
NAC-TRY (mg/L)	1.2 ± 0.6	3.8 ± 1.0	2.9 ± 2.3
NAC-5OH TRY (mg/L)	3.1 ± 2.7	n.d.	n.d.

## Data Availability

All data generated during this study are included in this article.

## Abbreviations

TRY	Tryptamine
NAC TRY	N-Ac Tryptamine
NAcTRP	N-Ac Tryptophan
NAcTRP-EE	N-Ac Tryptophan Ethyl Ester
5OH TRY	5-OH Tryptamine or Serotonin
NAC 5OH TRY	N-Ac-5-OH Tryptamine
5OH TRP	5-OH Tryptophan
TRP	Tryptophan
TEE	Tryptophan Ethyl Ester
TEE_IS1	TEE isomer 4.99
TEE_IS2	TEE isomer 5.39
AA	Anthranilic acid
3OH AA	3-OH Anthranilic acid
KYNA	Kynurenic acid
KYN	Kynurenine
3OH KYN	3-OH Kynurenine
MEL MEL ISO4.8	Melatonin or N-Ac-5- OCH3 Tryptamine Melatonin isomer (Rt 4.8 min)
